# Adolescent girls’ and young mothers’ knowledge and use of antenatal care in the Ahafo Region, Ghana: A cross-sectional study

**DOI:** 10.4102/phcfm.v16i1.4259

**Published:** 2024-07-09

**Authors:** Joseph Tabiri, Patience Adzordor, Vitalis Bawontuo, Shamsu-Deen Ziblim, Gugu G. Mchunu, Julian D. Pillay, Desmond Kuupiel

**Affiliations:** 1 Department of Public Health, Faculty of Health and Allied Sciences, Catholic University of Ghana, Fiapre, Ghana; 2 Yamfo College of Health, Yamfo, Ahafo Region, Ghana; 3 UNiTED Projects, Kpando, Ghana; 4 Department of Health Services Management and Administration, School of Business, SD Dombo University of Business and Integrated Development Studies (SDD-UBIDS), Wa, Ghana; 5 Department of Population and Reproductive Health, School of Public Health, University of Development Studies, Tamale, Ghana; 6 Faculty of Health Sciences, Durban University of Technology, Durban, South Africa; 7 Discipline of Public Health Medicine, School of Nursing and Public Health, University of KwaZulu-Natal, Durban, South Africa

**Keywords:** adolescents, young women, mothers, women, pregnancy care, awareness, use, antenatal care

## Abstract

**Background:**

Antenatal care (ANC) is crucial to reducing maternal and neonatal deaths, but few studies examined adolescent girls’ and young women’s ANC utilisation and knowledge in Ghana.

**Aim:**

To assess adolescents’ and young mothers’ knowledge of ANC, utilisation and factors influencing its use in Ghana.

**Setting:**

Tano North Municipality, Ahafo Region.

**Methods:**

This community-based, cross-sectional study involved 440 adolescent and young mothers (between 10 and 24 years). A structured questionnaire was employed to collect data face-to-face. Descriptive and statistical analyses were performed, and *p* < 0.05 was considered statistically significant.

**Results:**

Of the 440 respondents, most were aged 20–24 years (61.2%), married (30.0%), Christians (78.2%), completed junior high school (JHS) (47.8%) and traders (38.9%). Postnatal mothers were 71.6% (315), and all had utilised ANC services. Antenatal care knowledge was good among 75% (330) respondents, with no significant variation by age. Religion influenced knowledge, with Muslims having lower knowledge. Antenatal care utilisation was high (> 50%) among those aged 15–19 years, married, Christians, JHS graduates and traders. Age, marital status and employment type significantly influenced ANC utilisation. Individuals in the age group 15–19 years and married women demonstrated higher odds of utilising ANC services. Casual workers and unemployed respondents were found to have lower odds of utilising ANC services compared to traders.

**Conclusion:**

Age, marital status, and employment type influenced ANC utilisation in the Ahafo Region. Adolescent mothers under 15 years had lower rates, requiring targeted interventions to improve pregnancy outcomes.

**Contribution:**

This study highlights the knowledge and factors influencing ANC use in Ahafo Region and adds to the existing research evidence on ANC.

## Introduction

In low- and middle-income countries, approximately 55% of unintended pregnancies among adolescent girls aged 15–19 years result in complications and unsafe abortions.^1^ Adolescent mothers (aged 10–19 years) face higher risks of eclampsia, puerperal endometritis and systemic infections, and their babies are at a higher risk of low birth weight, preterm birth and severe neonatal conditions compared to young women aged 20–24 years.^2^ Maternal mortality is highest among adolescent mothers under 15 years, and complications during pregnancy and childbirth are higher among adolescent girls aged 10–19 years compared to women aged 20–24 years.^2,3^ In Ghana, adolescent pregnancy remains a significant public health challenge, with 109 888 recorded cases in 2020 alone.^4^

Efforts to mitigate pregnancy complications and preventable deaths include the implementation of the Ghana Ministry of Health’s Reproductive, Maternal, Newborn, Child and Adolescent Health and Nutrition Strategic Plan 2020–2025 and the National Strategic Framework on Ending Child Marriage 2017–2026.^5,6^ The Ghana National Safe Motherhood Protocol emphasises timely initiation and adequate use of antenatal care (ANC) services, which provide essential benefits to pregnant women and their unborn children.^7^

Antenatal care plays a crucial role in promoting maternal and child health by providing essential care, monitoring and interventions during pregnancy.^8,9^ Antenatal care refers to the healthcare services provided to pregnant women during their pregnancy, with the goal of ensuring a safe and healthy pregnancy and promoting the well-being of both the mother and the unborn baby.^8,9^ Antenatal care involves a series of regular check-ups, screenings and interventions that aim to monitor the progress of the pregnancy, detect any potential complications, and provide appropriate care and support to the pregnant woman.^8,9^ During ANC visits, healthcare providers typically assess the mother’s overall health, including measuring vital signs, checking weight and blood pressure, and monitoring the growth and development of the foetus.^8,9^ They also conduct various tests and screenings to identify any underlying health conditions or potential risks, such as blood tests, urine tests and ultrasounds. To this end, the World Health Organization (WHO) 2016 guideline recommends ANC models with a minimum of eight contacts to reduce perinatal mortality and improve women’s experience of care. It recommends pregnant women to have their first contact in the first 12 weeks’ gestation, with subsequent contacts taking place at 20, 26, 30, 34, 36, 38 and 40 weeks’ gestation.^8^ Scientific evidence is essential to continually improve ANC utilisation especially for vulnerable populations such as adolescent girls and young women (AGYW). However, only a few studies have examined this population in other regions of Ghana, highlighting the need for further investigation on ANC utilisation.^4,10^ Consequently, there is a lack of comprehensive understanding regarding AGYW’s knowledge of and utilisation of ANC services in the Ahafo Region of Ghana. Again, AGYW in Ahafo might face unique challenges related to reproductive health, including early pregnancies and inadequate access to ANC services. Thus, this study aimed to fill this knowledge gap by assessing AGYW’s knowledge of ANC and utilisation in the region. Understanding the factors influencing ANC utilisation among AGYW is essential for developing targeted interventions aimed at improving maternal and child health outcomes in this population.

## Research methods and design

### Study area

The study was conducted in the Tano North Municipality which was formerly created as the Tano North District in December 2004 until it was elevated to municipal assembly status in April 2018. The municipality is located in the eastern part of Ahafo Region and has Duayaw-Nkwanta as its capital town. It has a total land area of 837.4 km^2^ and constitutes about 1.8% of the total land area of then Brong Ahafo Region. The population of the municipality stood at 95 575 with a density of 95.5 persons per square kilometre for which the inhabitants are mostly made up of a wide range of ethnic groups comprising of Akans Ashantis, Bonos and Akuapems. In the municipality, three main religions are practiced: Christianity, Islam, and African Traditional Religion. The majority are Christians (78.6%), followed by Muslims (13.8%), and African Traditional Worshippers (0.8%). The healthcare setting of the Municipality comprises 10 community health planning and services (CHPS) compounds, 6 health centres and 1 hospital which serves as a referral point of care.^11^

### Study design

The study was a community-based descriptive cross-sectional study design with a quantitative approach to investigate the knowledge and utilisation of ANC services among adolescents and young mothers.

### Study respondents

This study recruited 440 AGYWs between the ages of 10–24 years who were either pregnant or within 6 weeks postnatal. Mothers within 6 weeks of delivery are likely to have recent and relevant experiences regarding ANC utilisation. Their recollections of the care received, knowledge gained and factors influencing ANC attendance are likely to be accurate and detailed, hence their inclusion in this study. This study’s respondents were limited to adolescents and young girls residing in the Tano North Municipality for at least 2 years. Adolescent girls and young women who met this study’s eligibility criteria but were sick or mentally challenged and unable to give information at the time of the study were excluded.

### Sample size determination

The total population of the Tano District was 95 575. The proportion of women in fertility age (WIFA) is calculated from 24% (11):

$$\text{Thus }24\%\text{ of the entire population}=\frac{95\;574*24}{100}=22\;938.$$ [Eqn 1]

Using the Yamane’s formula (Yamane, 1967), the sample size was calculated as follows:

$$n=\frac{N}{1+N(e^{2})}=\frac{22\;938}{1+2293(0.05^{2})}=\frac{22\;938}{57.35}=399.9=400$$ [Eqn 2]

*n* = desired sample size

*N* = population size of the Tano North District

*e* = level of precision at 95% confidence interval.

A 10% (40) nonresponse rate was added and thus a sample of 440 was used for the study.

### Sampling techniques

Out of the five sub-municipalities of the Tano North Municipality, a simple random technique by lottery method was used to select four submunicipals for the study. The submunicipals were listed on pieces of paper, folded and put in box which was vigorously shaken. Each submunicipal was randomly picked after every shake until four was obtained. In each submunicipal, two communities were conveniently selected based on availability of road network and transport, and size of the community’s population. Thus, eight communities, namely Bomaa, Techere, Susanho, Dua Yaw Nkwanta, Tanoso, Susaanso, Yamfo and Ahyiayem were selected for the study. Next, households were systematically recruited from each community. In the process, the first house located at the researcher’s right-hand side upon entry in the community was conveniently selected. Subsequent house(s) were selected by counting two houses at an interval away from the first house, until the required sample was obtained in the community. In a house that contained more than a household, the names of the household heads were noted and a simple random by means of lottery was employed to obtain the household to be used.

Finally, at a selected household, all single respondents meeting the inclusion criteria were identified and recruited to participate in the study. Where two or more eligible respondents were available, only one was randomly selected using the Yes or No procedure. In such scenario, pieces of paper with a single ‘Yes’ were folded. The papers were always folded up to the number of eligible participants. An individual who selected the ‘Yes’ was selected and taken through the data collection process.

### Data collection instruments

A structured questionnaire was designed based on the study objectives in English. The instruments were close-ended and used to gather data on background characteristics of respondents, respondent’s knowledge on the benefits of ANC and respondent’s utilisation of ANC service, factors that influenced ANC utilisation. The questionnaires were administered by the researchers themselves.

### Data collection procedures

Data for this study were collected in October 2022 using a pilot-tested instrument which was administered face-to-face in the homes of the respondents after obtaining informed consent. While the questionnaire was issued to respondents who could read and write (literates) to complete, respondents who could not read or write (illiterates) were guided by the data collector in interpreting the question items in their language of understanding. In this scenario, the choice of responses of respondents was noted on the questionnaire as a reflection of the respondents’ own responses. The data collectors checked to ensure that all the applicable questions were answered before leaving the respondents’ home. Prior to the main data collection, the data collection instrument was pretested with 15 adolescents and young girls from two different communities. These communities were not part of the included communities, but the effects and challenges observed in the administration of the instruments guided the realignment of ambiguities and helped increase the reliability of the final data collected.

### Data analysis

Data collected were initially entered in Microsoft Excel, cleaned and coded. Data cleaning involved the identification of incomplete entries and cross-checking against original documents and corrected. Data were then imported onto Stata version 20.0 for analysis. Descriptive statistics involving the demographic characteristics were presented in the form of frequency tables and charts. Respondents’ responses to question items that seek to envisage their understanding of the benefits of ANC services were also presented using descriptive statistics such as percentages. Knowledge was measured as either poor (less than 50% score) or good (greater than 50% score). Similarly, utilisation was categorised as either low (less than 50% score) or high (greater than 50% score).

Again, the number of ANC visits made by the respondents was first recorded through verbal assertion and later verifications were made in their ANC record book to confirm the information they had already given. Antenatal care visits made by the pregnant women or a previously pregnant women were compared with the WHO standard in order to properly define their utilisation status. Antenatal care visits less than four were termed underutilisation whereas four or more visits were considered high utilisation as per the WHO standard. The number of ANC visits (either less than or more than four visits) was therefore calculated into percentages. Thus, total visits less than 50% was considered low or poor utilisation, whereas more than 50% was considered high utilisation.

Statistical analysis included chi-square test and regression analysis to establish a relationship between the demographic characteristics and ANC knowledge as well as utilisation. Analysis of variance (ANOVA) was conducted to examine the variation in ANC utilisation among different age groups (10–14 years, 15–19 years and 20–24 years) for both previously pregnant respondents (postnatal mothers) and the currently pregnant respondents. All *p*-values less than 0.05 were considered statistically significant. Additionally, Bartlett’s test for equal variances was performed to assess the assumption of equal variances among the age groups.

### Ethical considerations

Approval to conduct the study was sought from the Committee on Human Research, Publications and Ethics (Kwame Nkrumah University of Science and Technology, School of Medical Science and Komfo Anokye Teaching Hospital – CHRPE/AP/524/22). The purpose of the study was well explained to participants for which their consent to participate or withdraw at any time of the research was sought for. Participants were therefore given consent forms to endorse, and they were assured of confidentiality and anonymity of information obtained from them through the study. Thus, codes were used to represent their identities. Though the participants of this study comprise adolescents (10–19 years), consent was sought from parents or guardians of respondents aged below 18 years.

## Results

### Demographic characteristics of respondents

Table 1 depicts the demographic characteristics of this study’s respondents. Of the 440 respondents, the majority (61.2%) were aged between 20 and 24 years, married (30.0%), Christians (78.2%), junior high school (JHS) graduates (47.8%) and traders (38.9%). Approximately 72% (315) of them were postnatal mothers, and 36.8% of 125 currently pregnant respondents were within the first trimester.

**TABLE 1:** Demographic characteristics of respondents (*N* = 440).

Characteristic	*n*	%
**Age (years)**		
10–14	19	4.3
15–19	152	34.5
20–24	269	61.2
**Marital status**		
Single	161	36.6
Married	132	30.0
Divorced	11	2.5
Separated	19	4.3
Cohabitating	117	26.6
**Religion**		
Christianity	344	78.2
Muslim	85	19.3
Traditionalist	11	2.5
**Educational level**		
None	12	2.7
Primary	71	16.1
JHS	210	47.8
SHS	115	26.1
Tertiary	32	7.3
**Employment type**		
Trading	171	38.9
Civil servant	52	11.8
Casual worker	109	24.8
Unemployed	96	21.8
Student	12	2.7
**Parity**		
0–1	204	46.4
2–3	175	39.7
4–5	61	13.9
**Currently pregnant**		
Yes	125	24.4
No	315	71.6
**Age of current pregnancies (*n* = 125)**		
1–3 months	46	36.8
4–6 months	43	34.4
7–9 months	36	28.8

JHS, junior high school; SHS, senior high school.

### Knowledge level of antenatal care services

About 75% of the respondents had good knowledge on the benefits of ANC services compared to 25% with poor knowledge. The results showed no significant difference of the ANC knowledge by age groups. Of the 19 respondents aged between 10 and 14 years in the study population, 15 (79%) had good knowledge on the benefits of ANC services, followed by those in the 20–24 years group with 75.5% out of 269 respondents, and 74.3% out of 152 respondents for those who were in the 15–19 years age group (Table 2). Religion was significantly associated (OR 2.43; 95% CI: 1.12–5.29) with decreasing knowledge of benefits of ANC. For Muslim respondents, OR value of 0.42 (95% CI: 0.25–0.70) and *p*-value of 0.001 indicated a decreased likelihood of having knowledge of ANC benefits among Muslims compared to Christians. The OR for traditional worshippers is 0.47 (95% CI: 0.13–1.65), but a *p*-value of 0.238 suggests no significant association (Table 3).

**TABLE 2:** Knowledge level on antenatal care services by age groups (*N* = 440).

Variable	Poor knowledge (*N* = 110, 25%)		Good knowledge (*N* = 330, 75%)		Total (*N* = 440, 100%)		Chi-square	*P*-value
	*n*	%	*n*	%	*n*	%		
**Age (years)**							0.1975	0.906
10–14 years	4	3.64	15	4.53	19	4.3	-	-
15–19 years	39	35.45	113	34.14	152	34.5	-	-
20–24 years	66	60.6	203	61.33	269	61.2	-	-
**Marital status**							4.989	0.288
Single	35	31.8	126	38.2	161	36.6	-	-
Married	37	33.6	95	28.8	132	30.0	-	-
Divorced	5	4.6	6	1.8	11	2.5	-	-
Separated	3	2.7	16	4.8	19	4.3	-	-
Cohabitating	30	27.3	87	26.4	117	26.6	-	-
**Religion**							12.152	0.002
Christianity	73	66.36	271	82.1	344	78.2	-	-
Muslim	33	30.0	52	15.8	85	19.3	-	-
Traditionalist	4	3.64	7	2.1	11	2.5	-	-
**Educational level**							3.3332	0.504
None	2	1.82	10	3.0	12	2.7	-	-
Primary	22	20.0	49	14.8	71	16.1	-	-
JHS	53	48.18	157	47.6	210	47.7	-	-
SHS	28	25.45	87	26.4	115	26.2	-	-
Tertiary	5	4.55	27	8.2	32	7.3	-	-
**Employment type**							5.4636	0.243
Trading	48	43.64	123	37.3	171	38.9	-	-
Civil servant	11	10.0	41	12.4	52	11.8	-	-
Casual worker	32	29.09	77	23.3	109	24.8	-	-
Unemployed	17	15.45	79	23.9	96	21.8	-	-
Student	2	1.82	10	3.0	12	2.7	-	-
**Parity**							3.8281	0.147
0–1	60	54.55	144	43.6	204	46.4	-	-
2–3	37	33.64	138	41.8	175	39.8	-	-
4–5	13	11.82	48	14.6	61	13.8	-	-

JHS, junior high school; SHS, senior high school.

**TABLE 3:** Unadjusted logistic regression between the sociodemographic variables and respondents’ knowledge of antenatal care benefits.

Variable	Odds ratio	*p*-value	95% Confidence interval
**Age (years)**		
10–14	1	-	-
15–19	0.77	0.663	0.24–2.47
20–24	0.8	0.713	0.26–2.52
**Marital status**		
Single	1	-	-
Married	0.71	0.204	0.42–1.21
Divorced	0.33	0.081	0.10–1.15
Separated	1.5	0.558	0.41–5.33
Cohabitating	0.8	0.432	0.46–1.40
**Religion**		
Christianity	1	-	-
Muslim	0.423	0.001	0.25–0.70
Traditionalist	0.47	0.238	0.13–1.65
**Educational level**		
None	1	-	-
Primary	1.34	0.334	0.74–2.41
JHS	1.4	0.322	0.72–2.70
SHS	2.42	0.108	0.82–7.13
Tertiary	2.24	0.322	0.45–11.11
**Employment type**		
Trading	1	-	-
Civil servant	1.44	0.334	0.69–3.06
Casual worker	0.93	0.793	0.55–1.58
Unemployed	1.8	0.064	0.97–3.35
Student	1.94	0.405	0.41–9.16
**Parity**		
0–1	1	-	-
2–3	1.54	0.071	0.96–2.47
4–5	1.53	0.224	0.77–3.02

JHS, junior high school; SHS, senior high school.

### Antenatal care services’ utilisation among previously pregnant adolescent girls and young mothers

All (100%) respondents had used ANC services. Of the 315 respondents among previously pregnant adolescent girls and young mothers (postnatal mothers), 164 (61%) had more than four ANC visits compared to 105 (39%) who had either less or equivalent to four ANC visits. Of the 315 previously pregnant respondents, ANC utilisation was high among respondents aged 20–24 years (*n* = 117, 71.3%), married women (*n* = 57, 34.9%), Christians (*n* = 124, 75.9%), JHS graduates (*n* = 75, 46.1%), traders (*n* = 74, 44.6%) and parity of 0–1 (*n* = 72, 44.2%) (Table 4).

**TABLE 4:** Relationship between the sociodemographic variables and antenatal care utilisation among previously pregnant adolescent girls and young women.

Variable	Low utilisation (*N* = 105, 39%)		High utilisation (*N* = 164, 61%)		Total (*N* = 440, 100%)		Chi-square	*P*-value
	*n*	%	*n*	%	*n*	%		
**Age (years)**							30.7678	0.000
10–14	3	2.8	0	0.0	3	1.1	-	-
15–19	45	43.0	47	28.7	93	34.5	-	-
20–24	57	54.2	117	71.3	173	64.4	-	-
**Marital status**							29.6389	0.000
Single	54	51.2	44	27.1	98	36.4	-	-
Married	23	22.1	57	34.9	80	29.7	-	-
Divorced	3	3.5	3	1.9	6	2.2	-	-
Separated	5	4.7	6	4.1	11	4.1	-	-
Cohabitating	20	18.6	54	32.9	74	27.6	-	-
**Religion**							0.397	1.8495
Christianity	85	81.4	124	75.9	209	78.2	-	-
Muslim	17	16.9	34	20.9	51	19.3	-	-
Traditionalist	3	1.7	6	3.2	9	2.5	-	-
**Educational level**							4.1142	0.391
None	3	3.5	3	2.2	6	2.7	-	-
Primary	14	13.4	29	17.8	43	16.1	-	-
JHS	53	50.6	75	46.1	128	47.8	-	-
SHS	28	27.3	41	25.3	69	26.1	-	-
Tertiary	7	5.2	16	8.6	23	7.3	-	-
**Employment type**							13.1029	0.011
Trading	31	30.2	73	44.6	104	39.0	-	-
Civil servant	11	11.1	20	12.3	31	11.8	-	-
Casual worker	28	26.7	38	23.4	64	24.7	-	-
Unemployed	30	29.1	28	17.1	58	21.8	-	-
Student	5	2.9	5	2.6	10	2.7	-	-
**Parity**							4.4953	0.106
1	53	50.5	72	44.2	125	46.5	-	-
2–3	35	33.7	71	43.5	106	39.7	-	-
4–5	17	15.8	21	12.3	38	13.8	-	-

JHS, junior high school; SHS, senior high school.

Table 5 further indicates significant associations between ANC utilisation and variables such as age, marital status and employment type. The odds ratio of utilising ANC services for the age group of 15–19 years was 5.62. The 95% confidence interval ranges from 1.57 to 20.08, suggesting that individuals in this age group have higher odds of ANC service utilisation compared to the 10–14 years group. The odds ratio for the age group of 20–24 years was 12.23 with a 95% confidence interval of 3.47–43.11, indicating a substantially increased likelihood of ANC service utilisation for individuals in this age group. The odds ratio for ‘married women’ was 2.94, and the high *z*-score of 4.34 corresponds to a significant *p*-value. The 95% confidence interval ranges from 1.81 to 4.79, indicating that married individuals have significantly higher odds of ANC service utilisation compared to the single group. The odds ratio for ‘casual worker’ respondents was 0.59, indicating a lower likelihood of ANC service utilisation compared to the traders group. The *p*-value of 0.041 suggests a significant association. The odds ratio for ‘unemployed’ individuals was 0.40, indicating a significantly lower likelihood of ANC service utilisation.

**TABLE 5:** Unadjusted logistic regression between the sociodemographic characteristics of previously pregnant women and antenatal care service utilisation.

Variable	Odds ratio	*Z*	*p*-value	95% Confidence interval
**Age (years)**				
10–14	1	-	-	-
15–19	5.62	2.66	0.008	1.57–20.08
20–24	12.23	3.89	0.000	3.47–43.11
**Marital status**				
Single	1	-	-	-
Married	2.94	4.34	0.000	1.81–4.79
Divorced	0.99	−0.01	0.988	0.29–3.38
Separated	1.64	1.00	0.316	0.62–4.28
Cohabitating	3.16	4.41	0.000	1.90–5.26
**Religion**				
Christianity	1	-	-	-
Muslim	1.32	1.09	0.275	0.80–2.17
Traditionalist	1.82	0.87	0.382	0.47–6.98
**Educational level**				
None	1	-	-	-
Primary	0.68	−1.32	0.188	0.38–1.20
JHS	0.69	−1.16	0.247	0.37–1.29
SHS	1.22	0.43	0.665	0.49–3.06
Tertiary	0.48	−1.17	0.243	0.14–1.65
**Employment type**				
Trading	1	-	-	-
Civil servant	0.75	−0.85	0.393	0.39–1.44
Casual worker	0.59	−2.04	0.041	0.36–0.98
Unemployed	0.40	−3.49	0.000	0.24–0.67
Student	0.61	−0.82	0.412	0.18–2.00
**Parity**				
0–1	1	-	-	-
2–3	1.46	1.76	0.078	0.96–2.22
4–5	0.85	−0.55	0.584	0.48–1.51

JHS, junior high school; SHS, senior high school.

### Antenatal care services’ utilisation among current pregnant adolescent girls and young mothers

Of the 125 respondents who were still pregnant, 82 (65.6%) recorded low (≤ 4 visits), while 43 (34.4%) recorded high ANC utilisation (> 4 visit). Of the 125 current pregnant respondents, ANC utilisation was high among respondents aged 15–19 years (*n* = 23, 53.5%), those cohabiting (*n* = 20, 46.5%), Christians (*n* = 38, 88.4%), JHS graduates (*n* = 51.2%), traders (*n* = 12, 44.6%), unemployed (*n* = 12, 44.6%) and parity of 2–3 (*n* = 19, 44.2%) (Table 6).

**TABLE 6:** Relationship between the sociodemographic characteristics and utilisation of antenatal care services among current pregnant women.

Variable	Low utilisation (*N* = 82, 65.6%)		High utilisation (*N* = 43, 34.4%)		Total (*N* = 125, 100%)		Chi-square	*P*-value
	*n*	%	*n*	%	*n*	%		
**Age (years)**							3.3636	0.186
10–14	13	15.9	2	4.7	15	12.0	-	-
15–19	38	46.3	23	53.5	61	48.8	-	-
20–24	31	37.8	18	41.9	49	39.2	-	-
**Marital status**							12.425	0.014
Single	36	43.9	14	32.6	50	40.0	-	-
Married	24	29.3	7	16.3	31	24.8	-	-
Divorced	3	3.7	0	0.0	3	2.4	-	-
Separated	4	4.9	2	4.7	6	4.8	-	-
Cohabitating	15	18.3	20	46.5	35	28.0	-	-
**Religion**							0.405	1.8098
Christianity	66	80.5	38	88.4	104	83.2	-	-
Muslim	14	17.1	5	11.6	19	15.2	-	-
Traditionalist	2	2.4	0	0.0	2	1.6	-	-
**Educational level**							0.679	2.3076
None	3	3.7	0	0.0	3	2.4	-	-
Primary	14	17.1	7	16.3	21	16.8	-	-
JHS	43	52.4	22	51.2	65	52.0	-	-
SHS	19	23.2	11	25.6	30	24.0	-	-
Tertiary	3	3.7	3	7.0	6	4.8	-	-
**Employment type**							1.649	0.800
Trading	22	26.8	12	27.9	34	27.2	-	-
Civil servant	8	9.8	7	16.3	15	12.0	-	-
Casual worker	26	31.7	11	25.6	37	29.6	-	-
Unemployed	25	30.5	12	27.9	37	29.6	-	-
Student	1	1.2	1	2.3	2	1.6	-	-
**Parity**							0.631	0.729
1	36	4.9	16	37.2	52	41.6	-	-
2–3	34	41.5	19	44.2	53	42.4	-	-
4–5	12	14.6	8	18.6	20	16.0	-	-

JHS, junior high school; SHS, senior high school.

Age and marital status showed significant associations with ANC utilisation with *p*-values less than 0.05. The age group of 15–19 years (OR 3.93, 95% CI: 0.81–19.02) and *p*-value of 0.089 suggests a marginally significant association. Cohabitating (OR 3.43, 95% CI: 1.38–8.52), *z*-score of 2.65 with a corresponding significant *p*-value of 0.008 suggest a higher likelihood of current utilisation for this category of respondents (Table 7).

**TABLE 7:** Unadjusted logistic regression between the sociodemographic characteristics and utilisation level of current pregnant women.

Variable	Odds ratio	*Z* score	*p*-value	95% Confidence interval
**Age (years)**				
10–14	1	-	-	-
15–19	3.93	1.7	0.089	0.81–19.02
20–24	3.77	1.63	0.103	0.76–18.66
**Marital status**				
Single	1	-	-	-
Married	0.75	−0.54	0.589	0.26–2.13
Divorced	1	-	-	-
Separated	1.28	0.27	0.785	0.211–7.83
Cohabitating	3.43	2.65	0.008	1.38–8.52
**Religion**				
Christianity	1	-	-	-
Muslim	0.62	−0.85	0.393	0.27–1.86
Traditionalist	1	-	-	-
**Educational level**				
None	1	-	-	-
Primary	1.02	0.04	0.966	0.36–2.90
JHS	1.18	0.25	0.806	0.36–3.74
SHS	2	0.74	0.46	0.32–12.59
Tertiary	1	-	-	-
**Employment type**				
Trading	1	-	-	-
Civil servant	1.6	0.75	0.453	0.47–5.51
Casual worker	0.76	−0.5	0.617	0.27–2.10
Unemployed	0.88	−0.25	0.799	0.33–2.35
Student	1.83	0.42	0.678	0.11–32.00
**Parity**				
1	1	-	-	-
2–3	1.26	0.55	0.581	0.56–2.84
4–5	1.5	0.74	0.458	0.51–4.38

JHS, junior high school; SHS, senior high school.

### Analysis of variance for antenatal care services’ utilisation among age groups

Analysis of variance was conducted to examine the variation in ANC utilisation among different age groups for both previously pregnant group (postnatal mothers) and the currently pregnant group. For the previously pregnant group, the ANOVA suggested that there was a significant difference in mean ANC utilisation among the age groups of 10–14 years, 15–19 years and 20–24 years. This is supported by the significant *F*-value (11.93) as well as the probability (Prob > *F*) of <0.05. Additionally, Bartlett’s test for equal variances was performed to assess the assumption of equal variances among the age groups, but the result suggests that there is no significant difference in variances between the age groups (Online Appendix Table 1-A1). However, in the case of the currently pregnant group, the ANOVA results suggest that there were no significant differences in the mean ANC utilisation among the age groups of 10–14 years, 15–19 years and 20–24 years. This is supported by the nonsignificant *F*-value (0.98) and the *p*-value of 0.3774. The Bartlett’s test for equal variances reports *p* = 0.232. This result suggests that there is no significant difference in variances between the age groups for current ANC utilisation (Online Appendix Table 2-A1). The Bartlett’s test results in both previously and current pregnant groups imply that the assumption of equal variances is met in both cases.

### Respondents’ perceived factors affecting antenatal care services’ utilisation

The Online Appendix Table 3-A1 presents insights into the perceptions of 440 respondents regarding factors influencing ANC utilisation in the Tano North Municipality. Among the key findings, 35.2% of respondents recognised that unplanned pregnancy positively influences ANC utilisation, while 70.7% believed that poor knowledge of ANC services hinders utilisation. Financial constraints were identified by 50.0% of respondents as a negative factor affecting ANC utilisation. Additionally, cultural or religious factors, distance to healthcare centres and high ANC fees were acknowledged by varying percentages of respondents as barriers to ANC utilisation. Furthermore, 72.5% of respondents identified the poor attitude of health workers as a negative influence on ANC utilisation, and 38.4% acknowledged stigmatisation as a factor. Poor family and social support were perceived as hindrances by 31.1% of respondents. A notable finding was that 81.4% of respondents did not consider the influence of traditional birth attendants as a significant factor affecting ANC utilisation, while 18.6% held the opposing view.

## Discussion

This was a cross-sectional study designed to report on AGYW’s knowledge of ANC, utilisation of ANC services, and factors influencing utilisation of ANC in Ahafo Region, Ghana. The results showed that most (75%) of the 440 respondents had good knowledge on the benefits of ANC services. The analysis indicated that identifying as Muslims was associated with lower odds of having good knowledge of ANC benefits. Similar findings were reported in Saudi Arabia,^12,13^ Egypt^14^ and Burkina Faso.^15^ In Saudi Arabia, Alharbi et al. and Al Mutairi et al. in their studies among pregnant women attending primary healthcare centres in Medina and Riyadh, respectively, reported that Muslim women had lower knowledge scores regarding ANC compared to women of other religions.^12,13^ Anukriti et al. examined the association between religion and maternal health knowledge in rural Egypt and found that Muslim women had lower knowledge scores compared to women of other religions.^14^ Agadjanian et al. also examined the association between religion and women’s health knowledge and behaviour in Burkina Faso and found that Muslim women had lower knowledge levels regarding reproductive health compared to women of other religions.^15^

The lower knowledge scores among Muslim AGYW in this study compared to those of other religions regarding ANC benefits could be attributed to a variety of factors such as cultural and societal factors, limited educational opportunities, language and communication barriers, a lack of health promotion programmes and limited engagement with healthcare providers.^16,17,18^ For instance, certain cultural and societal factors within Muslim communities may influence the dissemination of information and access to education about ANC.^17^ These factors can include gender norms, traditional beliefs, and restrictions on women’s mobility and autonomy, which may limit their opportunities to seek and receive information about ANC services. Also, Muslim women, particularly in certain regions or countries, may have limited access to education, resulting in lower literacy rates and reduced exposure to formal education. This lack of educational opportunities can contribute to lower knowledge levels about ANC benefits and healthcare in general. Besides, language barriers can hinder effective communication and understanding of healthcare information. In regions where Muslim women primarily speak languages other than the dominant language used in healthcare settings, the availability of translated materials or interpreters may be limited, leading to difficulties in accessing and comprehending information about ANC. Furthermore, in some areas, health promotion programmes and interventions may not effectively target Muslim communities or may not adequately address the specific cultural and religious considerations that influence health-related knowledge and behaviours. This lack of tailored programmes may contribute to lower knowledge levels among Muslim women. Moreover, Muslim women may face barriers to accessing healthcare facilities or may have limited interactions with healthcare providers due to cultural or religious norms. This limited engagement can result in missed opportunities for receiving information and education about ANC benefits directly from healthcare professionals. Nonetheless, it is important to note that these are potential reasons and should be interpreted with caution. The reasons for lower knowledge scores among Muslim women may vary across different contexts, regions and individuals. Further in-depth research focusing on AGYW who are Muslims in Ghana might help to gain a more comprehensive understanding of the underlying factors contributing to these disparities on knowledge of the benefits of ANC.

This study also found that high ANC utilisation (>4 visits) was recorded among previously pregnant respondents (61% out of 315) compared to current users (34.4% out of 125). This low ANC utilisation recorded among current users, perhaps, could be attributed to the fact that almost 37% (46 out of 125) were still within the first trimester, which connotes a bias to these study findings. This study’s analysis suggested that age, marital status and employment type were important factors associated with ANC utilisation among previously pregnant women in the Tano North Municipality, while only cohabitating was significantly associated with higher likelihood of high ANC utilisation compared to single women. Young age (≥15 years), being married or cohabitating, and engaging in trading are associated with higher odds of high ANC utilisation. Several studies have shown that older age is associated with higher odds of high ANC utilisation.^19,20,21,22^ Young women may have better awareness of the importance of ANC and greater access to healthcare services compared to adolescent younger girls of age less than 15 years.^4,19,20,21,22^ They may also be more likely to be married or in stable relationships, which can positively influence their utilisation of ANC services.^4,19,20,21,22^ Also, being married or in a cohabitating relationship has been consistently associated with higher ANC utilisation.^23,24^ Married or cohabitating women often have better social support systems and may be more likely to receive encouragement from their partners to seek ANC.^23,24^ They may also have better access to financial resources and transportation, which can facilitate their utilisation of ANC services.^4^ Moreover, engaging in trading or having regular employment has been linked to higher odds of high ANC utilisation.^4,25,26^ Women with stable employment may have access to health insurance or other financial resources that enable them to afford ANC services.^25,26,27^ Additionally, their regular work routines may allow for easier scheduling of ANC visits and fewer barriers to accessing healthcare facilities.^25,26,27^ To improve ANC utilisation among adolescent girls in Ghana, particularly Tano North Municipality, this study recommends youth-friendly healthcare facilities that are nonjudgmental, confidential and respectful of adolescents’ privacy, and community-based interventions to engage parents, teachers and community leaders to support ANC utilisation among adolescents. We also recommend engaging adolescents in decision-making to empower them to actively participate in decisions about their pregnancies, ANC visits and birth planning; peer support and mentorship to help reduce stigma, improve knowledge and encourage ANC utilisation among adolescents; integration of ANC education and awareness programmes within school curricula; and targeted campaigns and media outreach programmes for adolescent girls on the benefits of early and regular ANC visits.

Regarding the respondents’ perceived factors affecting ANC utilisation, this study found that about more than half of the 440 respondents suggested poor attitude of health workers (72.5%) and poor knowledge of ANC services (70.5%); and half indicated financial constraints (50.0%) as factors influencing ANC utilisation. While the respondents’ suggestion of poor attitude of health workers emphasises the importance of addressing interpersonal aspects, such as respectful and supportive care, to encourage women to seek ANC services, their suggestion of poor knowledge of ANC services might highlight the importance of improving awareness and understanding of ANC services among pregnant women in the Tano North Municipality. Regarding the current maternal healthcare policy in Ghana, ANC services are free in the country. Hence, financial constraints being a factor of low ANC utilisation as indicated by half of this study’s respondents suggests that the cost of ANC services might be a significant barrier for many women in accessing care which requires urgent attention. Transportation cost and clinical investigations cost as well as distance to access points might need further attention considering that approximately 34.7% of the respondents perceived distance to healthcare centres as a barrier to ANC utilisation as well as previous studies that evaluated the geographic access to ANC services in Ghana.^28,29,30,31,32^ These perceived factors provide insights into the challenges faced by women in the Tano North Municipality when accessing ANC services. However, understanding these barriers can inform the development of interventions and strategies to address them, ultimately improving ANC utilisation and maternal health outcomes in the region. It is important to note that these perceptions are subjective and may not necessarily reflect the actual factors influencing ANC utilisation, warranting further investigation and exploration through qualitative and quantitative research methods.

This study has several strengths. The study addresses an important and specific topic by focusing on AGYW’s knowledge and utilisation of ANC services. This targeted approach allows for a deeper understanding of the unique challenges and needs of this population. The use of a cross-sectional study design allows for data collection at a single point in time. This design can provide valuable insights into the status of knowledge and utilisation of ANC services among AGYW in the Ahafo Region. This study employed probability sampling methods and achieved a representative sample, hence the findings can be generalised to the larger population of AGYW in the Ahafo Region. The use of quantitative data collection methods, such as structured questionnaires, provided a standardised and easily quantifiable data. This facilitated the statistical analysis and allowed for comparisons and generalisations. Despites these strengths, this study has numerous limitations, and we caution that the results must be interpreted with careful consideration. The study relies on self-reported data, which may be subject to recall bias or social desirability bias. Respondents may provide answers they perceive as socially acceptable or may have difficulty accurately recalling information. Also, the study’s findings may not be fully representative of the entire population of AGYW in the other regions in Ghana, because approximately 10.5% (46 out of 440) were still within the first three months of pregnancy. Factors such as the specific sample characteristics or sampling methods employed may limit the generalisability of the findings. Cross-sectional studies provide a snapshot of a specific point in time and cannot capture changes or trends over time. Longitudinal data would be needed to assess knowledge and utilisation patterns and explore causal relationships more thoroughly. A cross-sectional study design may not allow for in-depth exploration of the reasons behind the findings or the experiences and perspectives of AGYW. Supplementing the study with qualitative methods, such as interviews or focus groups, could provide richer insights into the underlying factors influencing knowledge and utilisation of ANC services. Nonetheless, the findings of this study can inform policy and programme development aimed at improving knowledge and utilisation of ANC services among AGYW. This can contribute to better maternal and child health outcomes in the Ahafo Region towards the United Nations Sustainable Development Goal which aims to reduce maternal mortality to less than 70 per 100 000 live births by the year 2030.

## Conclusion

This study in the Ahafo Region, Ghana, examined the knowledge and utilisation of ANC services among AGYW. The findings revealed that while most respondents had good knowledge of ANC benefits, Muslim women had lower knowledge scores compared to women of other religions. Additionally, the study identified age, marital status, employment type and cohabitation as factors associated with ANC utilisation among previously pregnant women. Older age, being married or cohabitating, engaging in trading and having regular employment were linked to higher odds of high ANC utilisation. The study also highlighted perceived factors affecting ANC utilisation, such as the poor attitude of health workers, poor knowledge of ANC services, financial constraints and distance to healthcare centres. The findings provide insights into the challenges faced by women in accessing ANC services and emphasise the need for targeted interventions, including youth-friendly healthcare facilities, community-based interventions, improved awareness programmes and addressing financial barriers in the study area.
